# Synchronization Transition of the Second-Order Kuramoto Model on Lattices

**DOI:** 10.3390/e25010164

**Published:** 2023-01-13

**Authors:** Géza Ódor, Shengfeng Deng

**Affiliations:** Centre for Energy Research, Institute of Technical Physics and Materials Science, P.O. Box 49, H-1525 Budapest, Hungary

**Keywords:** synchronization, hybrid phase transition, criticality, chaoticity

## Abstract

The second-order Kuramoto equation describes the synchronization of coupled oscillators with inertia, which occur, for example, in power grids. On the contrary to the first-order Kuramoto equation, its synchronization transition behavior is significantly less known. In the case of Gaussian self-frequencies, it is discontinuous, in contrast to the continuous transition for the first-order Kuramoto equation. Herein, we investigate this transition on large 2D and 3D lattices and provide numerical evidence of hybrid phase transitions, whereby the oscillator phases $\theta_{i}$ exhibit a crossover, while the frequency is spread over a real phase transition in 3D. Thus, a lower critical dimension $d_{l}^{O}=2$ is expected for the frequencies and $d_{l}^{R}=4$ for phases such as that in the massless case. We provide numerical estimates for the critical exponents, finding that the frequency spread decays as $∼t^{-d/2}$ in the case of an aligned initial state of the phases in agreement with the linear approximation. In 3D, however, in the case of the initially random distribution of $\theta_{i}$, we find a faster decay, characterized by $∼t^{-1.8(1)}$ as the consequence of enhanced nonlinearities which appear by the random phase fluctuations.

## 1. Introduction

Synchronization within interacting systems is an ubiquitous phenomenon in nature. It has been observed in biological, chemical, physical, and sociological systems. Much effort has been dedicated to developing a theoretical understanding of its general features [1,2,3]. A paradigmatic model of *N* globally coupled oscillators was introduced and solved in the stationary state in the limit N→∞ by Kuramoto [4] and the macroscopic evolution of the system was later shown to be governed by a finite set of nonlinear ordinary differential equations [5]. An interesting property of the so-called first-order Kuramoto model is that it has a continuous phase transition, with a diverging correlation size, separating a synchronized phase from an unsynchronized one. Due to the chaoticity, emerging from nonlinearity, it obeys a scaling theory which is analogous to stochastic systems at the critical point and the whole set of critical exponents are known [4,5,6,7]. The corresponding universality class is termed as mean-field since, due to the all-to-all coupling, the individual oscillators interact with a mean-field of the rest of the oscillators. A challenging research direction aims to study the possibility and nature of the synchronization transitions in extended systems, where oscillators are fixed at regular lattice sites of finite dimension *d* and the interaction, in the extreme case, is restricted to that of nearest neighbors [6,8,9,10].

The so-called second-order Kuramoto model was proposed to describe power grids, analogous to the swing equation of AC circuits [11]. This is the generalization of the Kuramoto model [4] with inertia. One of the main consequences of this inertia is that the second-order phase synchronization transition—observed in the mean-field models of the massless first-order Kuramoto models—turns into a first-order one [12].

However, in lower dimensions, this has not been studied systematically. In [13], the numerical integration on 2D lattices suggested crossover transitions, with hysteresis in the case of the phase-order parameter. This means that the steady-state values depend on the initial conditions. Note that, due to the inherent heterogeneity of the quenched external torques $\omega_{i}^{0}$, proportional to the self-frequencies of the nodes, rare-region effects may occur, leading to frustrated synchronization and chimera states [13,14,15,16].

As real power grids are connected via complex networks, topological heterogeneity is also present, which can smear a phase transition, strengthening possible rare-region effects. However, even if topological heterogeneity is not present, it has still not yet been proven whether the massive model exhibits real phase transitions at low dimensions. Only conjectures, wherein the massive model has the same lower and upper critical dimensions as the first-order Kuramoto model (in the case of single peaked self-frequency distribution), are available. Accordingly, the mean-field phase transition for d≥4 of the phase-order parameter and a crossover below it [2] should occur (however, even the $d_{c}=4$ conjecture is debated, some studies concluded $d_{c}=5$ or higher [9]).

Thus, the upper and lower critical dimensions may be identical: $d_{c}=d_{l}=4$.

For the frequency entrainment of the massless model, the lower critical dimension is expected to be at $d_{l}^{O}=2$, similarly to the Mermin–Wagner theorem [17] for the planar XY spin model, which is supported by finite size scaling analysis [9]. Thus, for intermediate dimensions 2<d<4, real, nontrivial continuous phase transitions should occur. Analogously, for the massive case [13,16,18], entrainment phase transition is also expected for dimensions 2<d<4, as a very recent power-grid study [16] has indicated it for networks with graphs dimensions 2<d<3.

This has recently been published for the high-voltage power-grid networks of the USA and Europe and now we shall investigate it in the cases of pure 2D and 3D lattices, using finite size scaling. In another work [16], the linear approximation, which is expected to be valid for large couplings, provided a frequency spread decay law $\Omega ∼t^{-d/2}$. Now, we test the applicability of this approximation at the phase transition points.

Besides the dynamical scaling, the frequency order parameter exhibited a hysteresis and a discontinuity [16], which is known in statistical physics [19] as hybrid or mixed-type phase transition, for example, at tricriticality [20,21], or in other nonequilibrium systems [22,23,24]. Now, we investigate in detail this transition, which arises by the inertia in the Kuramoto model and results in hysteresis as we change the synchronization level of the initial states.

## 2. Models and Methods

### 2.1. The Second-Order Kuramoto Model

The time evolution of power grid synchronization is described by the swing equations [25], set up for mechanical elements with inertia. It is formally equivalent to the second-order Kuramoto Equation [11], for a network of *N* oscillators with phases $\theta_{i}\left(t\right)$:

(1) $$\begin{array}{ccc} \dot{\theta_{i}}\left(t\right) & = & \omega_{i}\left(t\right)\\ \dot{\omega_{i}}\left(t\right) & = & \omega_{i}^{0}-\alpha \dot{\theta_{i}}\left(t\right)+K\sum_{j=1}^{N}A_{ij}\sin\left[\theta_{j}\left(t\right)-\theta_{i}\left(t\right)\right]\;. \end{array}$$

Here, α is the damping parameter, which describes the power dissipation or an instantaneous feedback [18]; *K* is the global coupling related to the maximum transmitted power between nodes; and $A_{ij}$ is the adjacency matrix of the network which contains admittance elements. The quenched external drive, denoted by $\omega_{i}^{0}$, which is proportional to the self-frequency of the *i*-th oscillator and carries a dimension of inverse squared time $\left[1/s^{2}\right]$, describes the power in/out of a given node when Equation (1) is considered to be the swing equation of a coupled AC circuit; however, here, we chose it to be a zero-centered Gaussian random variable as the rescaling invariance of the equation allows to transform it within a rotating frame. For simplicity, one can assume that $\omega_{i}\left(0\right)$ is drawn from the same distribution as $\omega_{i}^{0}$ and numerically set $\omega_{i}\left(0\right)=\omega_{i}^{0}$, amounting to taking [s] = 1.

In our present study, the following parameter settings were used: the dissipation factor α was chosen to be equal to 0.4 to meet the expectations for power grids with the [1/s] inverse time physical dimension assumption. For modeling instantaneous feedback or an increased damping parameter, we also investigated the α=3.0[1/s] case, similarly to the work performed in in [16,18].

To generally solve the differential equations, we used the adaptive Bulirsch–Stoer stepper [26], which provides more precise results for large *K* coupling values than the Runge–Kutta method. The solutions depend on the $\omega_{i}^{0}$ values and become chaotic, especially at the synchronization transition, and thus, to obtain reasonable statistics, we needed strong computing resources, using parallel codes running on GPU clusters. The corresponding CUDA code allowed us to achieve ∼100× speedup on GeForce GTX 1080 cards as compared to Intel(R) Core(TM) i7-4930K CPU @ 3.40 GHz cores. The details of the GPU implementation will be discussed in a separate publication [27].

We obtain larger synchronization if the initial state is set to be phase synchronized: $\theta_{i}\left(0\right)=0$, but due to the hysteresis, one can also investigate other uniform random distributions such as: $\theta_{i}\left(0\right)\in \left(0,2\pi \right)$. The initial frequencies were set as: $\dot{\theta_{i}}\left(0\right)=\omega_{i}^{0}$.

To characterize the phase transition properties, both the phase-order parameter R(t) and the frequency spread Ω(t), termed the frequency-order parameter, were studied. We measured the Kuramoto phase order parameter:

(2) $$z\left(t_{k}\right)=r\left(t_{k}\right)\exp i\theta (t_{k})=1/N\sum_{j}\exp[i\theta_{j}\left(t_{k}\right)]\;,$$

by exponentially increasing the sampling time steps:

(3) $$t_{k}=1+1.08^{k}\;,$$

where $0\leq r(t_{k})\leq 1$ gauges the overall coherence and $\theta (t_{k})$ is the average phase. Here, the value 1.08 was used as a compromise in sampling a sufficient rather than an excessive number of time steps in order to allow one to observe asymptotic scaling within reasonable storage allocation. However, the calculation of the derivatives was performed adaptively at small time steps via the Bulirsch–Stoer stepper. The set of Equations (1) was numerically solved for $10^{3}$–$10^{4}$ independent initial conditions, initialized by different $\omega_{i}^{0}$-s and different $\theta_{i}\left(0\right)$-s if disordered initial phases were invoked. Then, the sample averages for the phases

(4) $$R\left(t_{k}\right)=\langle r\left(t_{k}\right)\rangle$$

and for the variance of the frequencies

(5) $$\Omega \left(t_{k},N\right)=\frac{1}{N}\sum_{j=1}^{N}(\bar{\omega }\left(t_{k}\right)-\omega_{j}{\left(t_{k})\right)}^{2}$$

were determined, where $\bar{\omega }\left(t_{k}\right)$ denotes the mean frequency within each respective sample.

In the steady state, which we determined by the visual inspection of the mean values $R(t_{k})$, we measured the standard deviations σ(R) of the order parameters $R(t_{k})$ in order to locate the transition point by fluctuation maxima. However, the transition point for $\Omega (t_{k},N)$ is characterized by a sudden drop in the Ω(t→∞,N) or by an emergence of an algebraic decay of Ω(t) as we increase *K*. In the case of the first-order Kuramoto equation, the fluctuations of both order parameters show a maximum at the respective transition points [28]. For the second-order Kuramoto, only the $\sigma (R\left(t_{k}\right))$ seems to have a peak at $K_{c}^{\prime }$, while for $\Omega (t_{k},N)$, we located a different transition point $K_{c}$, where the saturation of the steady-state value changed to a decay in the t→∞ limit.

### 2.2. Linear Approximation for the Frequency Entrainment

In Ref. [16], we showed that, similarly to the first-order Kuramoto model, the frequency-order parameter (5) decays as $\Omega \propto t^{-d/2}$ on a *d*-dimensional lattice in the large-system size and large coupling constant limit [9]. By applying the linear approximation sin(x)∝x and casting the continuum second-order Kuramoto equations into the momentum space, the phase velocity [$\omega \left(x,t\right)\equiv \dot{\theta }\left(x,t\right)$] is obtained [16]

(6) $$\begin{array}{cc} \omega (k,t)= & e^{-\frac{1}{2}t\left(\alpha +\Delta \right)}[\omega \left(k,0\right)(\left(\Delta +2-\alpha \right)e^{\Delta t}\\ \; & +\alpha +\Delta -2)-2Kk^{2}\theta \left(k,0\right)\left(e^{\Delta t}-1\right)]/2\Delta \;, \end{array}$$

where $\Delta =\sqrt{\alpha^{2}-4Kk^{2}}$. Hence, in this linear approximation, given α>0, the factor $e^{-1/2\alpha t}$ always ensures a full frequency entrainment for all K>0. When an initial disordered condition is considered, say θ(x,0) is uniformly distributed over $\left(0,\theta_{\max}\right)$, one has $\langle \theta \left(x\right)\theta \left(x^{\prime }\right)\rangle =\theta_{\max}^{2}/4$, suggesting that $\langle \theta \left(k,0\right)\theta \left(k^{\prime },0\right)\rangle =\delta^{d}\left(k\right)\delta^{d}\left(k^{\prime }\right)$. Hence, in the linear approximation, disorder from the initial condition does not affect the frequency spread (note that 〈ω(k,0)θ(k,0)〉=0) and we have (the same as in Ref. [16]):

(7) $$\begin{array}{cc} \Omega (t)= & \frac{1}{L^{d}}\int d^{d}x\langle {\left[\omega \left(x,t\right)-\bar{\omega }\left(t\right)\right]}^{2}\rangle \\ = & C_{d}\int_{2\pi /L}^{\pi /a}dkk^{d-1}\frac{e^{-t(\alpha +\Delta )}}{4\Delta^{2}}[\alpha +\Delta -2\\ \; & +\left(\Delta -\alpha +2\right)e^{\Delta t}{]}^{2}\;, \end{array}$$

where $\bar{\omega }\left(t\right)$ denotes the spatial average of ω(x,t), while *a* and $C_{d}$ are the lattice spacing and the geometric factor, respectively. Note that due to the lack of external noises, for every single run, depending on the initial $\bar{\omega }\left(0\right)$, α, and the system size (but not *K*), the simulation results indicate that $\bar{\omega }\left(t\right)$ always transits into a finite value that is not too far away from $\bar{\omega }\left(0\right)$. However, it should decay exponentially according to the Equation (6) for infinite systems.

As shown in Ref. [16], Equation (7) gives rise to the $t^{-d/2}$ law for any K>0, which manifests a rapid cutoff for large couplings in a typical finite system. However, in the regime where a linear approximation is invalid, weak couplings fail to maintain a narrow frequency entrainment and Ω is bound to be stationary after some time. Hence, a frequency entrainment phase transition from a finite stationary Ω value to an infinitely decaying Ω is expected.

## 3. Synchronization Transition in 2D

We solved the system of Equations (1) on large square lattices with periodic boundary conditions for the linear sizes L=200,400,1000,2000. The lattice structure is reflected by choosing $A_{ij}$ equal to 1 for the nearest neighbors and equal to zero otherwise. The self-frequencies were chosen randomly from a zero-centered Gaussian distribution with unit variance. The order parameters were calculated by ensemble averages over many samples.

### 3.1. Frequency Entrainment Phase Transition

It is known that the frequency order parameter (5) decays as $\Omega \propto t^{-d/2}$ in the case of the first-order Kuramoto model in the large coupling limit if we start from a random initial state [9]. We have also shown that the same is true for the second-order Kuramoto model in the linear approximation in [16]. Now, we investigate this at the neighborhood of the frequency entrainment transition point.

**Figure 1:**
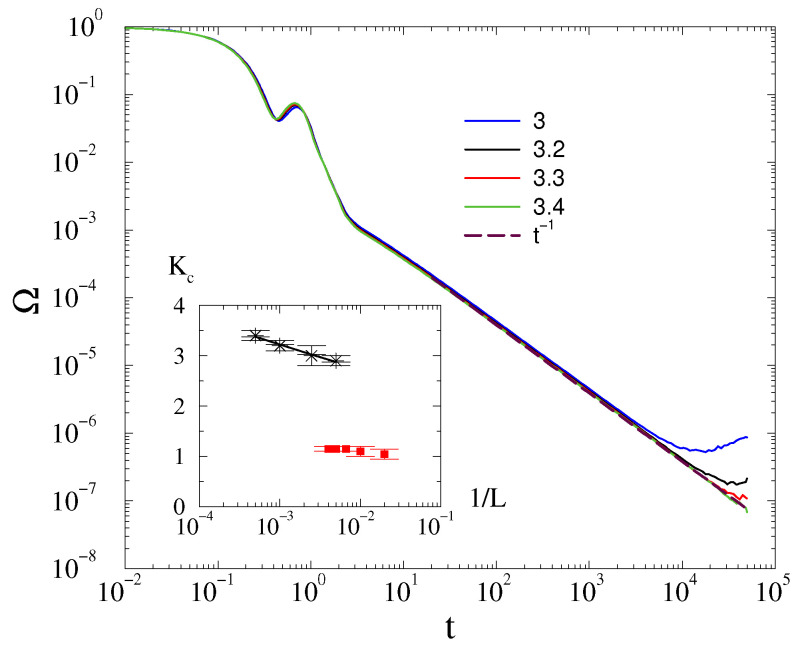
The frequency spread in 2D at α=3 for different *K* values, shown by the legends, for L=2000, in the case of ordered initial conditions. The dashed line marks a numerical fit at the critical point $K_{c}=3.4\left(1\right)$ with $t^{-d/2}$. Inset: the finite size scaling of the frequency entrainment transition point $K_{c}$ for various system sizes in 2D (black asterisks) and 3D (red boxes), for α=3 and under ordered initial conditions. One can see a logarithmic growth in 2D and a convergence to $K_{c}=1.15\left(5\right)$ constant value in 3D.

As Figure 1, shows the density decays as $\Omega \propto t^{-1}$ at the critical coupling strength, $K_{c}=3.4\left(1\right)$ in the case of ordered phase initial conditions, and for the α=3 damping factor. The decay behavior follows the same power law for $K\geq K_{c}$ before the finite size cutoff can take effect, and we see a saturation to finite values for $K<K_{c}$.

**Figure 2:**
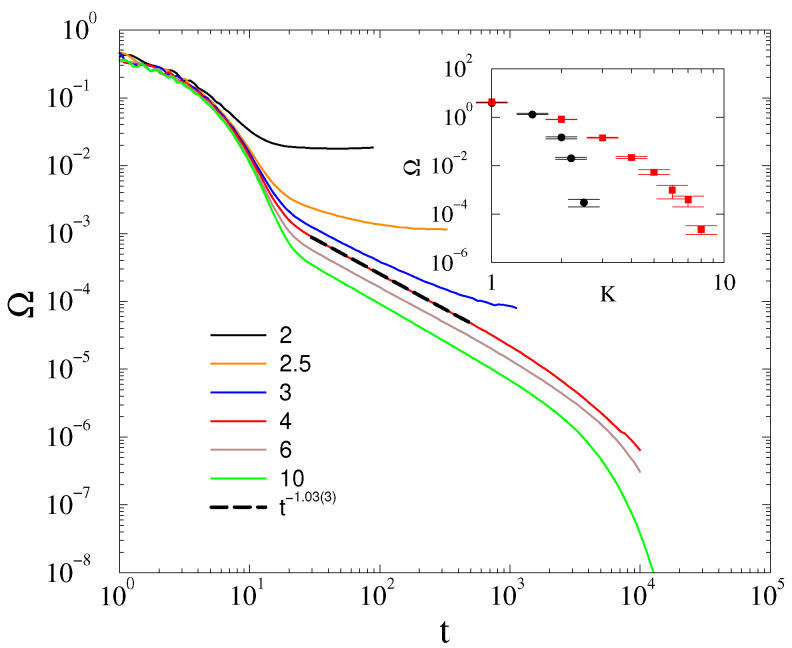
The frequency spread in 2D at α=0.4 for different *K* values, shown by the legends, for L=2000, using ordered initial conditions. The dashed line marks a numerical fit at the critical point $K_{c}=3.5\left(5\right)$ with $t^{-1.03(3)}$. Inset: Steady state values obtained by starting from ordered (black bullets) and disordered (red boxes) initial conditions.

The same is true for α=0.4: following a longer initial transient, we can see a decay at $K_{c}=3.5\left(5\right)$ characterized by $\Omega \propto t^{-1}$. as shown by Figure 2. An exponential finite size cutoff already occurs for t>1000 in contrast to the α=3 case, where this happened above $t>10^{4}$.

For smaller system sizes, the $K_{c}$-s do not move a lot, as we can see from the inset of Figure 1. The available data precision restricts finite size scaling, however, still we attempted it as shown in the inset of Figure 1. Assuming a logarithmic growth dependence, which is expected at the lower critical dimension [9], we obtained $K_{c}\left(1/L\right)\propto -1.7\left(1\right)\ln\left(1/L\right)$.

**Figure 3:**
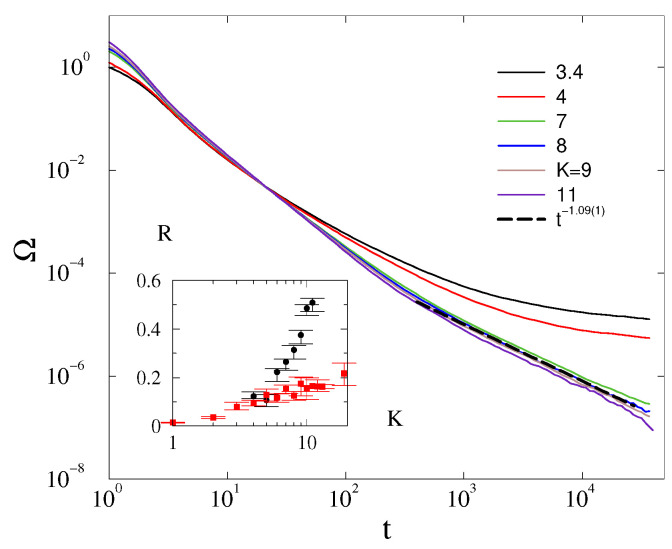
The frequency spread in 2D at α=3 for different *K* values, shown by the legends, for L=2000, in the case of disordered initial conditions. The dashed line marks a numerical fit at the critical point at $K_{c}=8.0\left(5\right)$ with $t^{-1.09(5)}$. Inset: Part of the hysteresis loop of *R* in 2D obtained by ordered (black bullets) and disordered (red boxes) initial conditions for α=3 and L=200.

**Figure A1:**
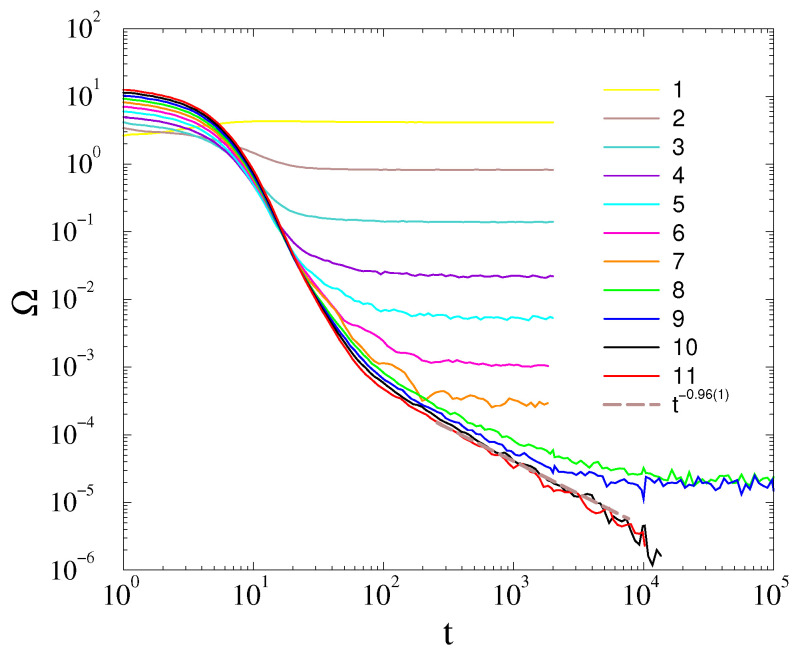
The frequency spread in 2D at α=0.4 for different *K* values, shown by the legends for L=2000, with disordered initial conditions. The dashed line marks a numerical fit at the critical point at $K_{c}=9.5\left(5\right)$ with $t^{-0.96(5)}$.

In the case of *fully disordered* initial conditions, $\theta_{i}\left(t=0\right)\in \left(0,2\pi \right)$, we found the same behavior as in case of the phase-synchronized starts, as one can see in Figure 3 for α=3 and Figure A1 for α=0.4 shown in the Appendix A.

The steady-state values, appearing for $t>10^{4}$ near the critical point of the α=0.4 damping factor case, are also determined and plotted in the inset of Figure 2 for L=200. We can see two branches, depending on the initial conditions. The upper branch corresponds to the disordered, the lower to the phase-ordered initial states. Thus, we can see a hysteresis-like behavior near the phase transition. However, the approach of $\Omega (K\to K_{c})$ is rather smooth, which is not surprising at a crossover point.

### 3.2. Phase-Order Parameter Transition

**Figure 4:**
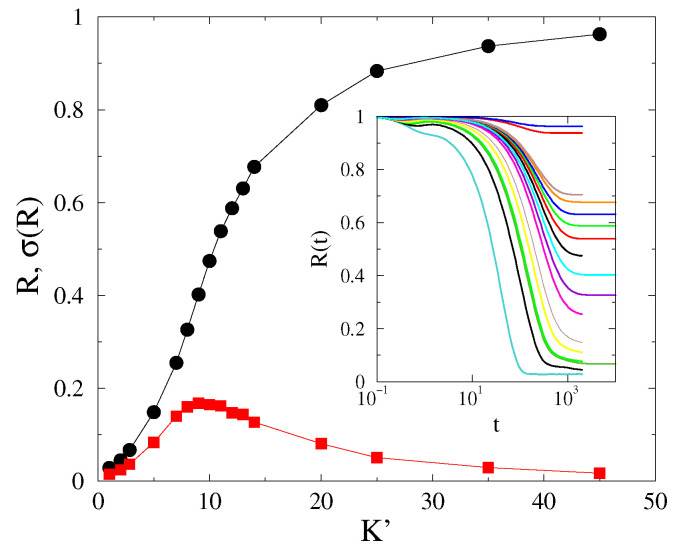
Steady -state Kuramoto order parameter (black dots) in 2D and its variance (red squares) at α=3 at different *K* values for L=200. Inset: R(t,L=200) for K= 1, 2, 3, 5, 7, 8, 9, 10, 11, 12, 13, 14, 20, 25, 35, 45 (bottom to top curves).

We determined the steady state values of R(t,L) by starting the systems from phase-coherent states up to $t_{max}=10^{4}-5\times 10^{4}$ followed by a visual inspection. For a certain system size *L*, we obtain the dependence of the stationary phase-order parameter $R_{\infty }$ on *K*. Figure 4 shows one such example for L=200 and α=3 in 2D. The transition point $K_{c}^{\prime }$ could then either be located by the peaks of σ(R), as chaoticity takes a maximum value at $K_{c}^{\prime }$ [16,28,29], or be estimated by the half value $R(L,K_{c}^{\prime })≃0.5$. However, this transition point did not coincide with the critical point $K_{c}$ determined by the order parameter Ω.

**Figure 5:**
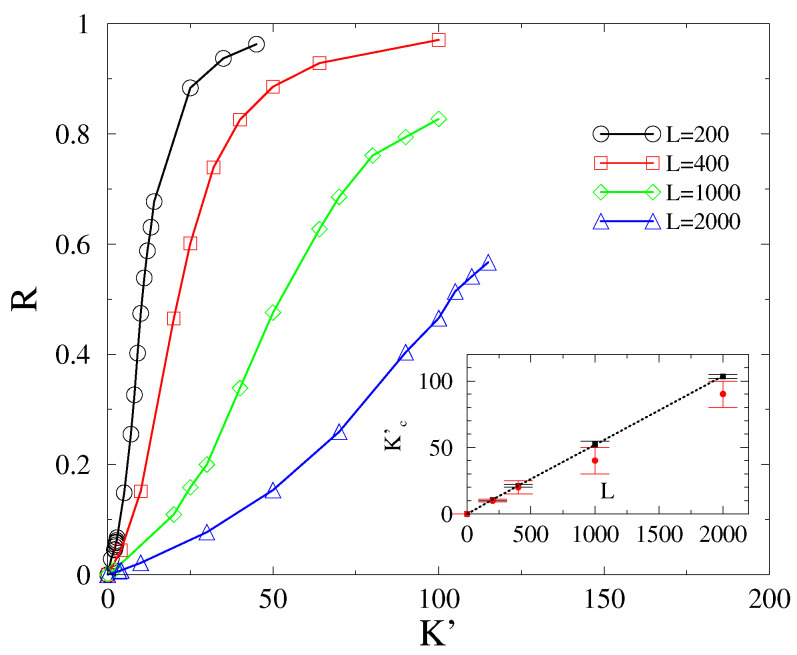
Finite-size behavior of *R* in 2D for α=3 and ordered initial conditions shows a crossover. Inset: finite-size scaling of $K_{c}^{\prime }$ as estimated by the half values of *R* (black boxes) and by the σ(R) peaks (red bullets) exhibit a linear growth.

As remarked in the Introduction, we conjecture that the Kuramoto-order parameter *R* exhibits a real discontinuous transition above $d_{l}^{R}>4$, while for $d\leq d_{l}^{R}$, a crossover transition ensues. To verify this conjecture, we estimate the transition point $K_{c}^{\prime }$ and check whether it diverges in an infinite system. The crossover transition nature (rather than a real transition) is immediately clear, as demonstrated by Figure 5, in which we see an evident shift in the transition point as the system size is varied. The σ(R) also became wider and wider as we increased the size.

Particularly, the inset suggests that the transition point shifts linearly with *L* in 2D [$K_{c}^{\prime }\left(L\right)\propto L$]. Hence, the transition points exhibit a power-law growth with exponents, suggesting that $K_{c}^{\prime }\left(L\right)\to 0$ as L→0 and $K_{c}^{\prime }\left(L\right)\to \infty$ as L→∞.

For disordered initial conditions, we can find much lower steady state values indicated by the inset of Figure 3. The hysteresis loop closes at very large *K* values only, as was also demonstrated in [16] for power-grid networks.

## 4. Synchronization Transition in 3D

In 3D, following the results of the first-order Kuramoto model we expect a real phase transition of the frequency-order parameter, but a crossover for the phases. Similarly to 2D, we solved the system of Equation (1) on large cubic lattices with periodic boundary conditions for following linear sizes L=50,100,150,200,250 in order to perform finite size analysis. Again, the interaction is described by choosing $A_{ij}$ equal to 1 for nearest neighbors and zero otherwise.

### 4.1. Frequency Entrainment Phase Transition

**Figure 6:**
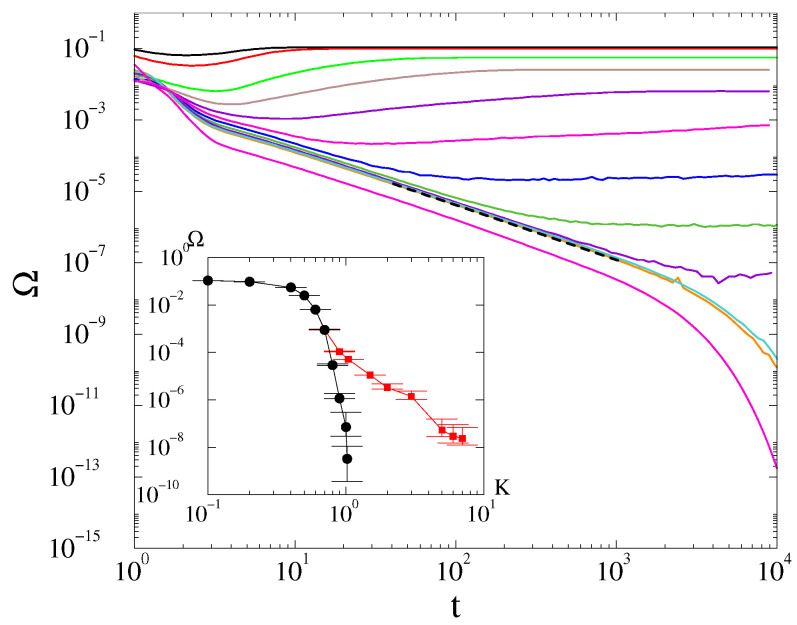
The frequency spread in 3D at α=3 for K= 0.1, 0.2, 0.4, 0.5, 0.6, 0.7, 0.8, 0.9, 1, 1.05, 1.1, 2 (top-to-bottom curves) for L=200 linear sized lattices and phase-ordered initial conditions. The dashed line marks a numerical fit at the critical point $K_{c}=1.02\left(2\right)$ with $t^{-d/2}$. Inset: Steady state values obtained by starting from ordered (black bullets) and disordered (red boxes) initial conditions.

In the case of *phase-ordered initial states*, the frequency spread decays with the law $\Omega \left(t\right)\propto t^{-d/2}$ above $K_{c}≃1.1$, followed by a finite-size cutoff as shown on Figure 6 for L=200. Doing the finite-size scaling of the transition point, we find that $K_{c}$ does not change within error margins for L≥150 and we estimate a finite value $K_{c}=1.15\left(5\right)$ as shown in the inset of Figure 1.

**Figure 7:**
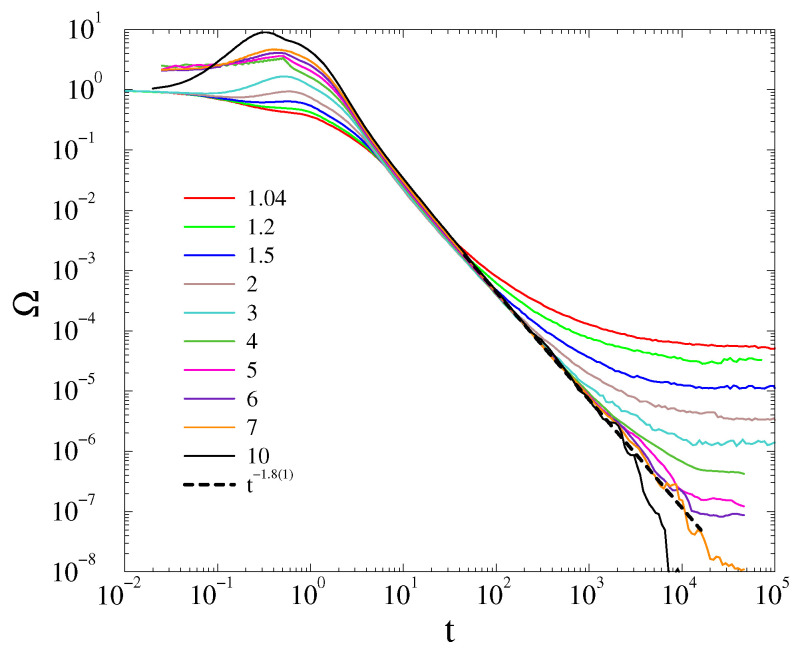
The frequency spread in 3D at α=3 for different *K* values, shown by the legends, for L=200 and disordered initial conditions. The dashed line marks a numerical fit at the critical point $K=K_{c}≃7$ with $t^{-1.8(1)}$.

However, in case of the *fully random phase initial condition*, the decay at the critical point seems to deviate from the $t^{-d/2}$ law. It can be fitted by $\Omega \left(t\right)\propto t^{-1.8(1)}$ at $K=K_{c}≃7$, as shown on Figure 7. Note, that around criticality, in the $t>10^{3}$ region, where finite-size effects emerge, the slopes of the curves increase, suggesting a nontrivial correction as in case of the first-order Kuramoto model [10]. Due to the limited computing power, this excludes the possibility of seeing a crossover towards a $\Omega \left(t\right)\propto t^{-d/2}$ asymptotic behavior obtained by the linear approximation. We investigated this behavior for the other levels of randomness in the initial state $\theta_{max}=1,\;1.75,\;1.9$, but only found it in the fully random phase case.

In the case of disordered initial conditions, the level-off of Ω(t), thus, $K_{c}$ also occurs at a much higher coupling, than in the ordered initialization case as the consequence of the phase transition. Therefore, we conjecture a possible different scaling behavior, if any, at the higher $K_{c}$ value. The steady-state behavior of Ω is also shown in the inset of Figure 6. At first sight, it may not suggest a discontinuous transition, but as we applied log-log scales to observe the rapid changes, two branches emerge and we can see the occurrence of a wide hysteresis loop as the consequence of different initial conditions.

### 4.2. Phase-Order Parameter Transition

**Figure 8:**
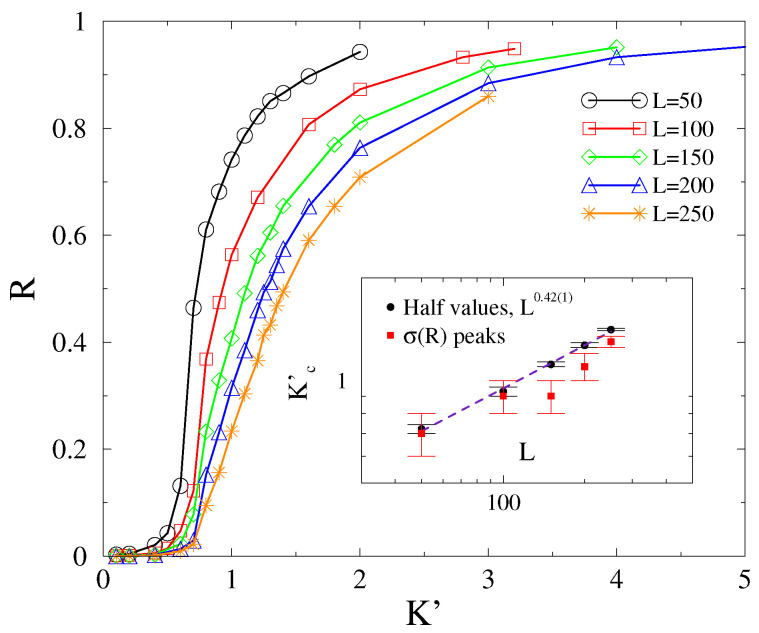
Finite-size behavior of *R* in 3D for α=3 and the ordered initial conditions, shows a crossover. Inset: finite-size scaling of $K_{c}^{\prime }$ as estimated by the half values of *R* (black bullets) as well as by the σ(R) peaks (red boxes) exhibit a power-law growth.

**Figure A2:**
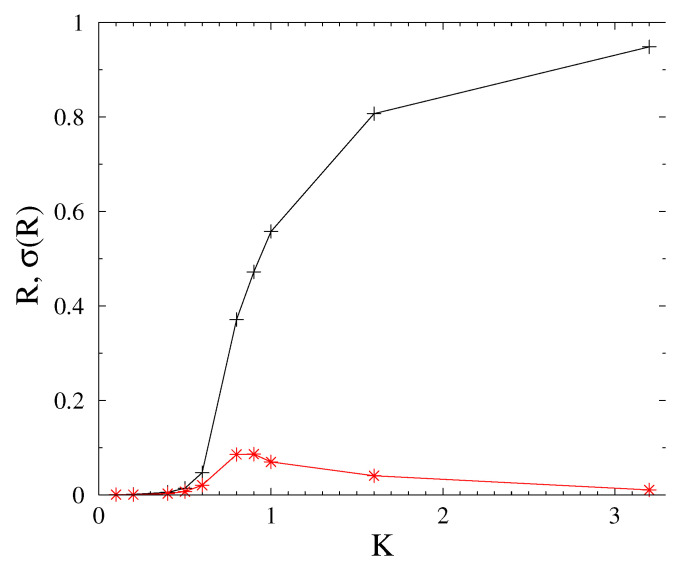
Steady state Kuramoto-order parameter (black ‘+’) in 3D and its variance (red ‘*’) at α=3 for different $K^{\prime }$ values for L=100. Inset: R(t,L=100).

We determined the Kuramoto-order parameter values in the steady state for cubes with linear sizes of L=50,100,150,200,150, using α=3 and ordered initial conditions. We display the results for L=100 in the Appendix A, as can be seen in Figure A2. We attempted a finite size scaling analysis as in 2D, as shown in Figure 8. The σ(R) distributions become very smeared as L→∞, making it difficult to locate the peaks. However, nonetheless, a reasonable power-law fit could be obtained, in agreement with the half-value method described in Section 3.2: $K_{c}^{\prime }\propto L^{0.42(1)}$, as one can see in the inset of Figure 1. Thus, we still find a crossover behavior in 3D, with a lower $K_{c}^{\prime }$ growth exponent than in 2D, which is expected to decrease as we increase the dimension approaching the lower critical dimension.

## 5. Conclusions

We performed an extensive numerical study of the synchronization transition of the second-order Kuramoto model in 2D and 3D. We provided numerical evidence that, while the phase-order parameter exhibits crossover transition, which diverges with the system size in a power-law manner, the frequency-spread-order parameter exhibits real phase transition in 3D. In the latter case, the finite size dependence of the critical point is negligible on the system sizes we investigated, and the transition point for an infinite system, estimated through extrapolation, is also very close to those measured in finite systems except for a logarithmic correction in 2D. However, the transitions of both order parameters exhibit hysteresis behavior, with the steady-state values, which depend on the initial conditions.

However, the variance of *R*, representing chaoticity over the initial self frequency choices, has a smeared peak around the crossover point, with a growing spread as we increase *L*. This makes the location of the crossover point difficult to determine, but we used an alternative method, using half values of *R*, consistently with the peak locations, as a reliable way of obtaining it. While the $K_{c}^{\prime }\left(L\right)$ grows linearly with *L* in 2D, in 3D, we found a nontrivial power-law dependence: $K_{c}^{\prime }\left(L\right)\propto L^{0.42(1)}$.

For the Ω order parameter, we did not find a peak at the critical point, in contrast with the case of the massless Kuramoto model, in agreement with a first-order type phase transition behavior. However, we found asymptotic power-law decay: $\Omega \left(t\right)\propto t^{-d/2}$ for $K\geq K_{c}$, which agrees with the linear approximation result. This allowed us to perform a crude finite size scaling of $K_{c}$, which exhibits a logarithmic growth of $K_{c}$ in 2D and a saturation in 3D. Thus, similarly to the massless Kuramoto [9], we claim $d_{l}^{O}=2$ for the lower critical dimension.

We also found a deviation from the linear approximation law in d=3 in case of disordered initial states: $\Omega \left(t\right)\propto t^{-1.8}$. This behavior might be the consequence of a slow crossover in time or the nonlinearities due to the phase fluctuations on the upper branch of the frequency-order hysteresis curve. This behavior may be observable in real-power grid situations, as we found in [16], in the case of larger damping factors. For α=0.4, this anomalous power-law region is less extended, but this is true for all the PL-s we see: the damping factor elongates the scaling regions in agreement with the rescaling invariance of the differential equation, as shown in [16].

The coexistence of power-law dynamics of Ω and the hysteresis in the steady states thus classifies this as a hybrid or mixed type of phase transition, which would be interesting to study further. 

## Data Availability

Data are available upon request from the corresponding author.

## Appendix A

In this appendix, we show results in 2D for the Ω(t) decay solution in the case of disordered initial conditions at α=0.4.

Furthermore, we also plot the steady-state behavior of *R* in 3D, for α=3, at L=100 and under ordered initial conditions. One can observe a peak in σ(R) at K≃0.85, where R≃0.5.
